# Glucoraphanin conversion into sulforaphane and related compounds by gut microbiota

**DOI:** 10.3389/fphys.2025.1497566

**Published:** 2025-02-10

**Authors:** Tetiana R. Dmytriv, Oleh Lushchak, Volodymyr I. Lushchak

**Affiliations:** ^1^ Department of Biochemistry and Biotechnology, Vasyl Stefanyk Precarpathian National University, Ivano-Frankivsk, Ukraine; ^2^ Research and Development University, Ivano-Frankivsk, Ukraine

**Keywords:** Glucoraphanin, gut microbiota, Nrf2, oxidative stress, sulforaphane

## Abstract

Glucosinolate glucoraphanin, common in cruciferous vegetables, is a biologically stable precursor of isothiocyanates, such as sulforaphane and erucin, potent activators of Nrf2 signaling coordinating an adaptive response to oxidative stress. Sulforaphane is formed by the hydrolysis of glucoraphanin by a plant enzyme called myrosinase, which is inactivated in the stomach of mammals. Since the latter do not have enzymes possessing myrosinase-like activity, glucoraphanin can be metabolized by the gut microbiota, to sulforaphane, sulforaphane-nitrile, glucoerucin, erucin, and erucin-nitrile. Emerging evidence suggests that variations in gut microbiota composition significantly influence the efficiency and outcome of glucoraphanin metabolism, while sulforaphane itself may reciprocally modulate gut microbiota composition and functionality. This review examines the bidirectional interactions between glucoraphanin, sulforaphane, and gut microbiota. We assume that sulforaphane alleviates intestinal inflammation and oxidative stress maintaining intestinal homeostasis and gut barrier integrity. Besides, the role of sulforaphane in breaking the vicious cycle of oxidative stress and gut dysbiosis is reported, demonstrating the potential of dietary isothiocyanates to support gut barrier function.

## 1 Introduction

Glucoraphanin (GRP) is a naturally occurring sulfur-containing compound mainly produced by cruciferous plants such as cauliflower, broccoli, radish, cabbage, arugula, kale, bok choy, Brussels sprouts, collards, and watercress. It belongs to biologically relatively inert glucosinolates (GLs), plant secondary metabolites originating from amino acids (Jones et al., 2006; Prieto et al., 2019). Glucoraphanin is the most abundant in raw broccoli (Xu et al., 2020) and arugula (Ku et al., 2016). In particular, screening of the profile and levels of GLs in 42 cultivars of *Brassica oleracea* Crops showed that the content of GRP varied from 0 to 141 µmol/100 g fresh weight with the highest amounts in broccoli (Verkerk et al., 1999).

Under certain conditions, biologically inert GLs can be converted into corresponding isothiocyanates (ITCs) and/or nitrile derivatives by the enzyme called myrosinase (β-thioglucoside glucohydrolase, EC 3.2.1.147). If ITCs such as sulforaphane (SFN) are widely known for their health benefits, ITC-nitriles are considered biologically inactive (Basten et al., 2002; Matusheski and Jeffery, 2001). In plants, myrosinases and GRP are located in different places, i.e., they are spatially separated to prevent inadvertent activation of the so-called mustard oil bomb (Angelino et al., 2015). The latter is a defense mechanism developed by cruciferous plants to prevent consumption by animals. The destruction of plant tissues by herbivores or humans makes GRP accessible for myrosinases. This results in the conversion of the GLs into corresponding biologically active ITCs with a pungent taste, that is the “detonation” of a mustard oil bomb (Vanduchova et al., 2019; Sikorska-Zimny and Beneduce, 2021a). In particular, GRP is converted into sulforaphane (SFN), a powerful health-promoting remedy with potent antioxidant and anti-inflammatory properties in animals and humans (Vanduchova et al., 2019; Sikorska-Zimny and Beneduce, 2021a). The formation of ITC/ITC-nitriles is controlled by a thermolabile epithiospecifier protein (ESP), which directs myrosinase-catalyzed hydrolysis of GLs to corresponding ITC-nitriles. ESP activity negatively correlates with the degree of SFN formation and depends on factors such as Fe^2+^ ions, temperature, and pH (Matusheski et al., 2006). If the formation of SFN-nitrile depends on ESP, then SFN is formed spontaneously. However, SFN is chemically unstable due to the reactive electrophilic carbon atom in the ITC group (N=C=S). Therefore, in plants, SFN is stored in the form of its inert precursor GRP, which is converted into SFN after the destruction of plant tissues (Janczewski, 2022). Thus, SFN as a biologically active compound, is virtually absent in an intact plant.

Cooking cruciferous vegetables at high temperatures such as boiling leads to a significant loss of myrosinase activity, decreasing the level of ITCs such as SFN (Oloyede et al., 2021). However, chewing raw cruciferous vegetables induces the release of active myrosinase in the oral cavity (pH 6.7–7.3) (Baliga et al., 2013; Sarvan et al., 2018). Plant myrosinase demonstrates optimal activity at pH from 5 to 9, whereas at lower pH it is partially or completely inactive (Andersson et al., 2009; Galádová et al., 2022). Thus, plant myrosinase consumed with food can potentially hydrolyze GRP into SFN or/and SFN-nitrile in the oral cavity with subsequent inactivation in the stomach (Evans et al., 1988). Therefore, in such cases, plant myrosinase probably does not provide the formation of therapeutically significant concentrations of SFN. However, it has been shown that mammalian gut microbiota can also convert GRP into SFN available to the host, probably with the participation of bacterial myrosinase (Lai et al., 2010; Li et al., 2011; Kellingray et al., 2014). Several reviews discuss the metabolic fate of dietary GLs (Bouranis et al., 2021b; Castro-Torres et al., 2020; Sikorska-Zimny and Beneduce, 2021b), but the role of the gut microbiota is often overestimated. There is a significant gap in the study of the metabolism of individual GLs and their subsequent transformation into biologically active compounds, which makes it difficult to assess the actual contribution of the gut microbiota. A significant influence of GLs/ITCs on the composition and functional activity of the gut microbiota has also been reported, but the potential molecular mechanisms involved are little discussed. In this review, we consider GRP hydrolysis by the gut microbiota with the production of SFN and other products, further interconversions of the metabolites, as well as the influence of GRP and its bioactive derivative SFN on gut microbiota and molecular mechanisms of action of dietary SFN to maintain a healthy gut microbiota.

## 2 Metabolism of glucoraphanin in the gastrointestinal tract: role of the gut microbiota

The simulation model of gastrointestinal enzymatic digestion of GRP showed that no mammalian digestive enzymes could hydrolyze GRP *in vitro* (Lai et al., 2010). In particular, the concentration of GRP did not change during oral/amylase digestion, gastric/HCl-pepsin digestion, and intestinal/pancreatin-bile digestion, demonstrating its indestructibility by the gastrointestinal enzymes. However, in the intestine, GRP becomes available as a substrate for gut microbiota.

The human gastrointestinal tract (GIT) is inhabited by a significant number of diverse microorganisms that, interacting with the host, form a dynamic community defined as the gut microbiota. The colon has the largest microbial load in the body and contains about 10^14^ bacteria with the dominant phyles of Firmicutes (renamed Bacillota), Bacteroidetes (Bacteroidota), Actinobacteria (Actinomycetota), and Proteobacteria (Pseudomonadota) (Afzaal et al., 2022; De Vos et al., 2022). The first two phyla represent 90% of gut microorganisms that constantly interact with the host, affecting its health. If shortly, the phylum Firmicutes consists of over 200 genera, including *Enterococcus*, *Lactobacillus*, *Ruminicoccus*, *Bacillus*, and *Clostridium*, representing 95% of the Firmicutes phylum. The second dominant phylum Bacteroidetes is mainly represented by the genera *Bacteroides* and *Prevotella* (Rinninella et al., 2019).

The microbial community provides numerous benefits for the host contributing to metabolism of nutrients, drugs, xenobiotics, maintenance of gut barrier function and intestinal immune homeostasis (Jandhyala et al., 2015). In addition, the gut microbiota has a significant genomic content, which complements the human genome promoting an effective symbiosis. In particular, several studies have shown that gut microbes convert GRP to ITCs and/or ITC-nitriles complementing host gastrointestinal enzymes in these processes. Generally, we have found only four published experimental studies related to gut microbiota-associated GRP hydrolysis. Table 1 summarizes information regarding the experimental conditions and the identified microbiota-produced GRP metabolites.

**TABLE 1:** Microbiota-produced glucoraphanin metabolites in *ex vivo* studies. Abbreviations and marks: GRP, glucoraphanin; SFN, sulforaphane.

No	Experimental conditions	Shown increased production of metabolites	References
1	Rat cecal microbiota from rats pre-treated with GRP (150 μmol/kg body weight) cultivated *ex vivo* with 0.5 mM GRP	SFN, erucin-nitrile	Lai et al. (2010)
2	Rat cecal microbiota from rats fed 10% cooked broccoli diet for 0–14 days cultivated *ex vivo* with 183 μM GRP	SFN, erucin	Liu et al. (2017)
3	Human fecal bacteria exposed to a repeated dose of GRP for seven 12-h cycles	Glucoerucin, SFN, SFN-nitrile	Kellingray et al. (2014)
4	Fecal microbiota from healthy individuals fed with standardised meal containing 200 g of cooked broccoli cultivated *ex vivo* with 50 μM GRP	Total isothiocyanates such as SFN and erucin	Li et al. (2011)

Lai et al., (2010) showed that cecal microbiota from rats administered with GRP (150 μmol/kg body weight) by gavage provides GRP hydrolysis *ex vivo*. In particular, GRP content decreased with time in the MRS medium (supports the growth of lactobacilli and others) containing 0.5 mM GRP. Microbiota-mediated hydrolysis of GRP was significantly greater after 12 and 24 h of the experiment. A similar pattern was seen in microbial hydrolysis of GRP in RCM medium (supports the growth of clostridia, bifidobacteria, and others) with 0.5 mM GRP, but only at a 12 h time point. SFN was found as a product formed only in MRS medium, and erucin nitrile — in all conditions (Lai et al., 2010). Liu and colleagues (2017) also demonstrated the myrosinase-like activity of the cecal microbiota from rats fed 10% cooked broccoli diet for 0–14 days. In particular, incubation of the cecal microbiota with an excess of GRP led to a time-dependent increase in the concentration of the ITCs, such as SFN and erucin. The microbial GRP hydrolyzing activity increased as rats were fed broccoli for longer periods. This indicates that the presence of GRP in the diet enhances rates of its microbial hydrolysis (Liu et al., 2017). However, some studies have reported significant conversion of GRP to glucoerucin. For example, in an experimental model where human fecal bacteria were exposed to a repeated dose of GRP for seven 12-h cycles a significant conversion of GRP to glucoerucin was found and to a lesser extent, it was metabolized to SFN, SFN-nitrile, and SFN-conjugates (Kellingray et al., 2014). In another study, Li and colleagues (2011) established myrosinase-like activity of fecal microbiota from healthy individuals fed with a standardized meal containing 200 g of cooked broccoli. Fecal microbiota from selected human excretes containing high or low content of ITCs was cultivated *ex vivo* with 50 μM GRP that resulted in GRP degradation of different intensities. In particular, bacteria from selected human excretions containing high ITC content were able to break down more GRP than bacteria from excretions with low ITC content. However, ITCs such as SFN produced by bacteria during incubation were unstable in the culture medium, so their final concentration was insignificant. A direct relationship between specific types of bacteria and GRP hydrolysis to ITCs was not established (Li et al., 2011). Nevertheless, in the absence of plant myrosinase, microbial metabolism of GRP is sufficient to raise SFN levels in the stool. In particular, Zhang et al. (2023) showed that consumption of steamed broccoli sprouts with inactivated plant myrosinase by mice promoted the appearance of SFN in feces. Additionally, it was found that consuming steamed broccoli increased SFN content throughout the GIT with the highest levels in the colon, where GRP levels were lowest (Zhang et al., 2023).

**FIGURE 1:**
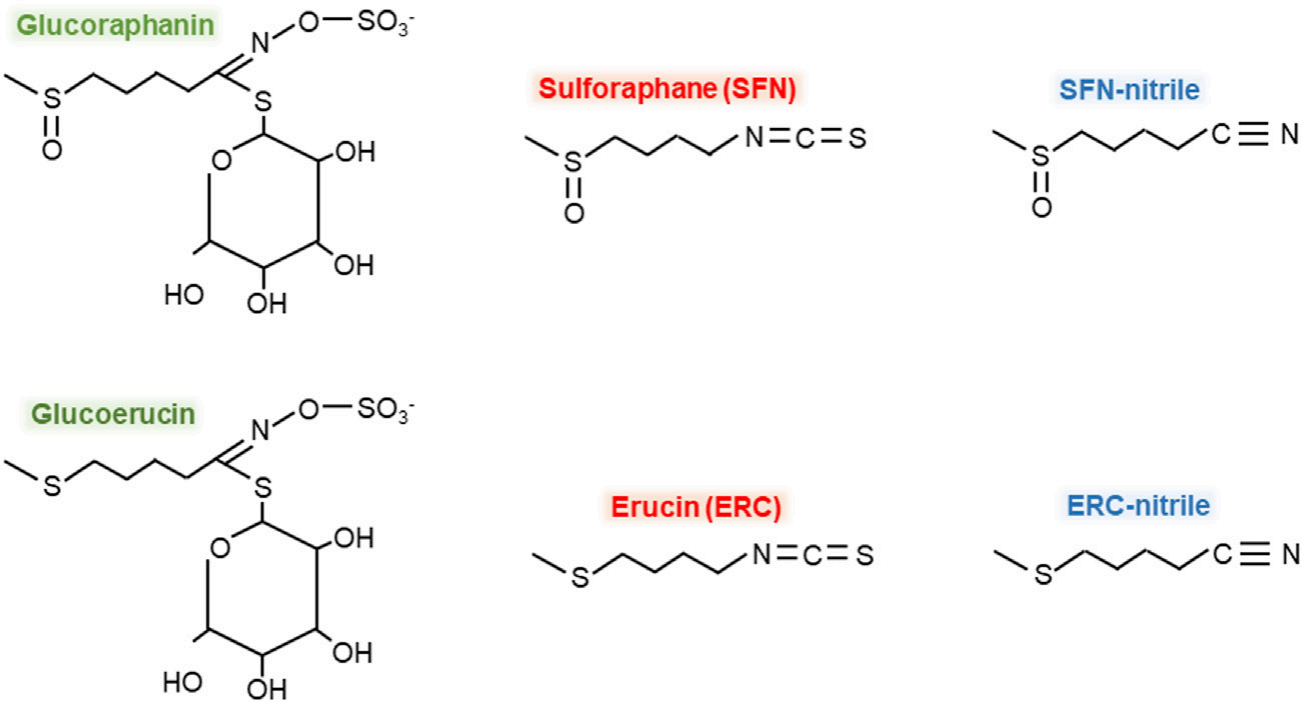
Chemical formulas of glucoraphanin and its microbiota-produced metabolites. Green color – glucosynolates, red – isothiocyanates, blue – isothiocyanate-nitriles.

Metabolites of GRP produced by microbiota can be categorized into the following three groups: (i) GLs such as glucoerucin, (ii) ITCs such as SFN and erucin, and (iii) ITC-nitriles such as SFN-nitrile and erucin-nitrile. Figure 1 shows the structural formulas of GRP and the above-mentioned microbiota-produced GRP metabolites. However, to date, information on microbiota-associated GRP metabolism is insufficient. For example, it is not clear whether the gut microbiota can produce SFN-conjugates such as glutathione SFN-conjugate (SFN-GSH) or SFN-N-acetylcysteine conjugate (SFN-NAC) or identified compounds are produced by human cells.

Intragastric administration of GRP (172 mg/kg body weight) to germ-free and human microbiota-associated (HMA) mice led to the excretion of approximately 30% unchanged GRP in the urine of both germ-free and HMA mice (Budnowski et al., 2015). This indicates that some part of GRP is not metabolized and excreted intact. Bheemreddy and Jeffery (2007) found 5% intact GRP when rats were administered 150 μmol/kg GRP purified from broccoli seed. Total urinary excretion was 20% of the oral dose, including 12.5% SFN-NAC, 0.65% free SFN, 2% SFN-nitrile, and 0.1% erucin. In contrast, neither GRP nor its metabolites were detected in feces (Bheemreddy and Jeffery, 2007). These data clearly show that catabolism of GRP in the gut depends on many factors and the type of consumed by animals or humans food may play a crucial roles here.

**FIGURE 2:**
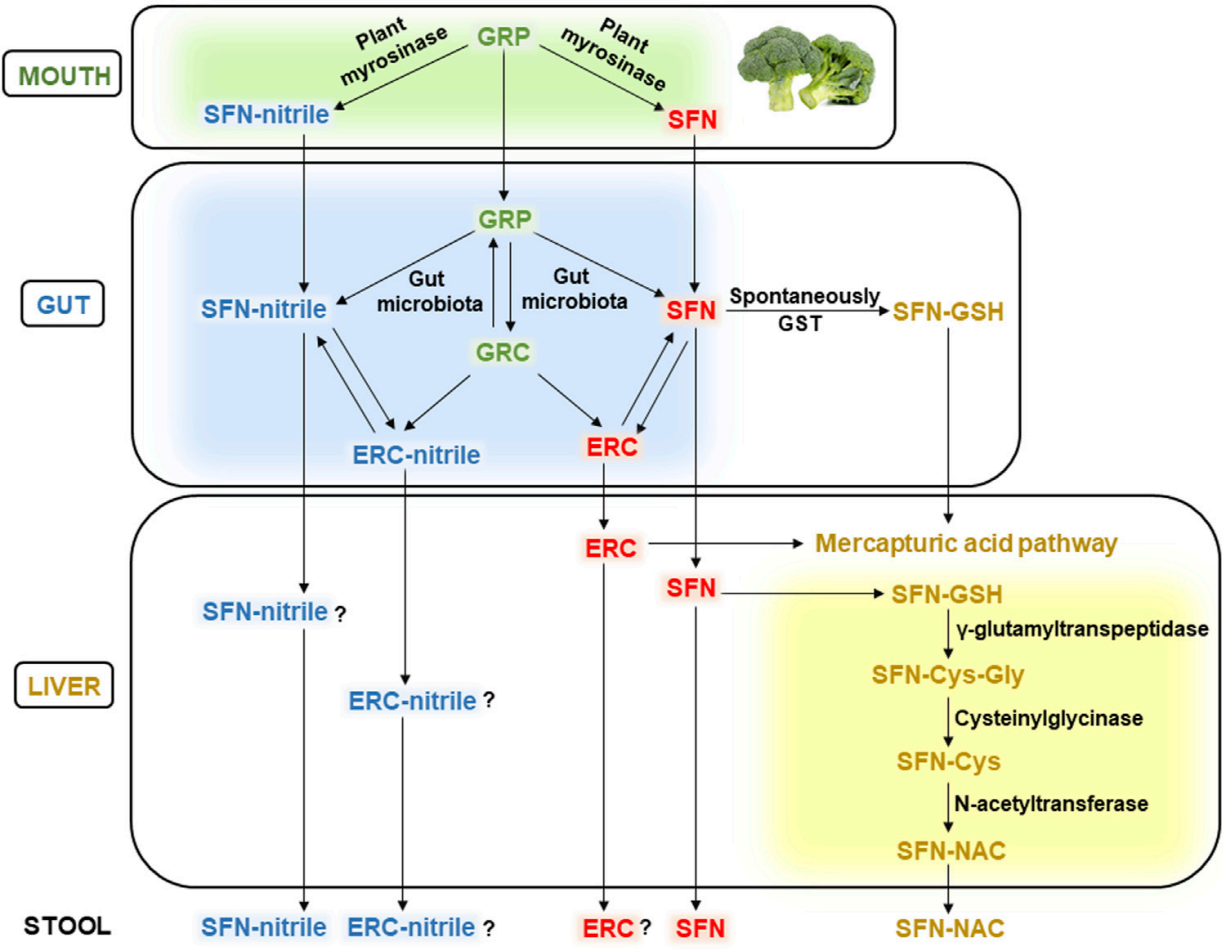
Metabolism of glucoraphanin in the human body. Abbreviations: GRP, glucoraphanin; SFN, sulforaphane; GRC, glucoerucin; ERC, erucin; GST, glutathione-S-transferase; SFN-GSH, SFN-glutathione conjugate; SFN-Cys-Gly, SFN-cysteinglycine conjugate; SFN-Cys, SFN-cystein conjugate; SFN-NAC, SFN-N-acetylcysteine conjugate. See the text for details.

Figure 2 schematically demonstrates the metabolism of GRP and its biologically active derivative SFN within the GIT. As mentioned above, chewing cruciferous vegetables such as broccoli makes GRP available for plant myrosinase causing the conversion of GRP to SFN and/or SFN-nitrile in the oral cavity (Sarvan et al., 2018). In the intestine, GRP is metabolized by the gut microbiota to SFN and/or SFN-nitrile, probably by bacterial myrosinase, or converted to glucoerucin, that can be hydrolyzed to erucin and/or erucin-nitrile. In addition, GRP and glucoerucin, SFN and erucin, as well as SFN-nitrile and erucin-nitrile can be interconverted (Charron et al., 2018).

Microbiota-produced GRP metabolites are absorbed in the intestine and transported with blood to various organs, including the liver, and partially excreted with feces. Generally, they are metabolized through the mercapturic acid pathway. The initial stage can occur already in the intestine due to the spontaneous interaction between electrophilic carbon in the SFN molecule and nucleophilic thiol group of GSH, forming the SFN-GSH conjugate. In addition, intestinal glutathione-S-transferase (GST) can catalyze the same reaction (Gu et al., 2022). In the liver, the formed SFN-GSH conjugates enter the mercapturic acid pathway producing SFN-cysteinglycine (SFN-Cys-Gly) in the reaction catalyzed by γ-glutamyltranspeptidase enzyme. Cysteinylglycinase splits off the glycine from SFN-Cys-Gly conjugate, forming the SFN-Cys. The latter is converted to SFN-NAC by N-acetyltransferase, transported with the blood to the kidneys and excreted in the urine (Vanduchova et al., 2019; Bouranis et al., 2021b).

Gut microbiota-produced erucin is metabolized similarly to SFN, as shown in Figure 2. However, the detoxification pathways of erucin-nitrile and SFN-nitrile are poorly studied (the question marks in Figure 2). In addition, if free SFN, SFN-nitrile, and SFN-NAC were identified in the stool (Bouranis et al., 2024), erucin and erucin-nitrile were not mentioned (as far as we know). Potentially, this can be associated with the excretion of minor concentrations of erucin and its nitrile, which are difficult to identify.

The study of the distribution of SFN metabolites in plasma, urine, and stool at different time points (24, 48 and 72 h) (Bouranis et al., 2024) after a single serving of broccoli showed that: (i) in plasma, SFN-nitrile was the only detected metabolite and accordingly dominant at all time points, (ii) in urine, SFN-NAC was the main metabolite between 0 and 3, 3-6, and 6–24 h, while SFN-nitrile was the dominant metabolite between 24-48 and 48–72 h, (iii) free SFN accounted for more than 95% of detected metabolites in the stool after 48 and 72 h, while after 24 h, free SFN was the major metabolite, followed by SFN-NAC and SFN-nitrile. A study by Bouranis et al. (2021a) demonstrated a similar to the above-mentioned urinary excretion pattern of SFN metabolites. In particular, SFN-NAC reached its peak 6 h after participants consumed a single dose of broccoli sprouts containing 200 µmol of SFN equivalents, and between 12 and 48 h, its level decreased continuously. However, the excretion of SFN-nitrile increased with a peak at 6 h in six of ten participants and a peak between 24 and 48 h in four participants (Bouranis et al., 2021a). Clearly, the qualitative composition and levels of GRP metabolites may vary among individuals, probably due to the difference in the composition and metabolic activity of the gut microbiota.

To date, a positive association between the representatives of the gut microbiota and SFN metabolism has been established. For example, Bouranis et al. (2024) found that members of the genus *Dorea*, *Bifidobacterium*, and *Ruminococcus torques* are positively associated with SFN metabolite excretion, while members of the genus *Blautia* and *Alistipes*, on the contrary, are negatively associated (Bouranis et al., 2024). Indeed, some bacterial strains carry out myrosinase-like activity in the GIT. In particular, Holman and colleagues (2023) identified 309 bacterial sequence variants associated with the expression of myrosinase-like enzymatic activity, which included genera such as *Lactococcus*, *Bifidobacterium*, *Lactobacillus*, *Bacteroides*, *Pseudomonas*, *Staphylococcus*, *Enterococcus*, and *Streptomyces*. (Holman et al., 2023). However, GRP as well as SFN can also affect the gut microbiota composition and functionality. For example, the presence of GRP in the diet facilitated its microbial hydrolysis rates, i.e., the intestinal bacteria of individuals who consume more GLs potentially can hydrolyze more GRP (Liu et al., 2017). In the next section, the influence of GRP and SFN on the gut microbiota will be highlighted.

## 3 The influence of glucoraphanin and its bioactive derivative sulforaphane on the gut microbiota

The diet is a crucial factor determining the functionality of gut microbiota and its qualitative and quantitative composition. An individual microbial pattern can promote health or be detrimental. For example, an imbalance of the gut microbiota, called dysbiosis or dysbacteriosis, is associated with the development of metabolic syndrome, cardiovascular disease, inflammatory bowel disease, and neurological disorders. So, we are what we eat (Zmora et al., 2019).

Consumption of cruciferous vegetables rich in GLs/ITCs modulates the gut microbiota composition affecting the host’s health. Table 2 shows the effect of GRP and SFN on the gut microbiota in several recent studies. For example, consumption of GRP at a dose of 150 μmol/kg body weight for 6 weeks increased the richness and diversity of bacterial species in C57BL/6 mice consuming a high-fat diet (HFD) (Xu et al., 2020). In particular, GRP significantly altered the community structure of the gut microbiota, increasing the abundance of Bacteroidetes and decreasing Firmicutes in comparison with the HFD group who did not consume GRP (Xu et al., 2020). A study by Bankole and colleagues (2024) established a similar effect in C57BL/6 mice who consumed HFD containing 1% broccoli seed extracts with 0.13% GRP and 0.322% mustard powder with plant myrosinase. The percentage of Firmicutes and Bacteroidetes were 87.2% and 11.4% in the HFD group but were 80.4% and 15.9% in the HFD group consuming GRP. In addition, a significant decrease in phylum Verrucomicrobiota but an increase in phyla Actinobacteriota and Deferribacterota in GRP-treated mice were found (Bankole et al., 2024).

**TABLE 2:** The effect of glucoraphanin and sulforaphane on the gut microbiota. Abbreviations: GRP, glucoraphanin; SFN, sulforaphane. Marks: ↑ – increased, ↓ – decreased.

No	Experimental model	Effect on the gut microbiota	References
1	C57BL/6 mice consumed a high-fat diet with 150 μmol/kg body weight GRP	Phyla Bacteroidetes ↑ and Firmicutes ↓	Xu et al. (2020)
2	C57BL/6 mice consumed high-fat diet containing 1% broccoli seed extract with 0.13% GRP	Alpha diversity ↑, Phyla Firmicutes and Verrucomicrobiota ↓, Phyla Bacteroidetes, Actinobacteriota, and Deferribacterota ↑	Bankole et al. (2024)
3	Hyperuricemic Sprague-Dawley rats treated with 10 mg/kg mixture of GRP and myrosinase by oral gavage	Firmicutes/Bacteroidetes ratio ↑, *Bacteroides cellulosilyticus* and *Clostridium boltea* ↓	Wang et al. (2023)
4	43 commensals and pathogens from human fecal or gastrointestinal biopsy samples cultivated with 10 μM SFN at 0.1% oxygen	No significant changes were found, however, 55% of the isolates showed a tendency to increased growth. Under 0.01% oxygen, *Escherichia coli* ECE2348/69 showed a significant increase in growth at 20 μM SFN. However, in aerobic conditions (21% oxygen), SFN at concentrations of 5 μM–20 μM inhibited its growth	Marshall et al. (2023)
5	Autism spectrum disorders-like rats intraperitoneally injected with SFN 20 mg/kg	Bacteroidetes and Actinobacteria ↑, genera *Prevotella, Peptostreptococcus*, and *Oribacterium* were positively correlated with SFN treatment	Yang et al. (2023)
6	C57BL/6 mice with ulcerative colitis intragastric administered with 20 mg/kg SFN	Phyla Bacteroidetes ↑ and Firmicutes ↓, Firmicutes/Bacteroidetes ratio ↑	Zhang et al. (2020)

The ratio between the two dominant phyla Firmicutes/Bacteroidetes (F/B) is often defined as a potential biomarker for a variety of disorders. As mentioned above, GRP consumption can lead to a decrease in the abundance of Firmicutes and an increase in Bacteroidetes, contributing to a downward shift in their ratio. On the contrary, an increase in the F/B ratio can be considered a risk factor for the development of obesity. For example, Koliada and colleagues (2017) showed that obese individuals have a significantly higher level of Firmicutes and lower level of Bacteroidetes compared to normal-weight or lean adults. Indeed, the F/B ratio increased with increasing body mass index, confirming the relationship between gut microbiota and obesity (Koliada et al., 2017). Thus, the consumption of cruciferous vegetables or GRP/SFN-rich preparations can potentially prevent obesity and affecting the gut microbiota may be the mechanism responsible. It is important to note that microbiota composition is subjected to seasonal variation. For example, Actinobacteria were more abundant and Bacteroidetes were less abundant in summer-derived samples compared to those obtained during other seasons, whereas Firmicutes content was seasonally independent (Koliada et al., 2020). Seasonal variation was supposed to be connected with extensive consumption of fruits and vegetables in summer time.

Marshall et al. (2023) studied the effect of SFN on the growth of 43 human common commensals and pathogens. In particular, in aerobic conditions at 21% O_2_, SFN inhibited the growth of enteropathogenic *E. coli EPEC ECE2348/69* demonstrating antimicrobial properties. However, in anaerobic conditions at 0.01% O_2_, SFN had the opposite effect. It increased the anaerobic respiration of *Escherichia coli EPEC ECE2348/69*, promoting its growth (Marshall et al., 2023). This indicates that a significant part of the results obtained in aerobic investigations should not be translated with expected *in vivo* results, since they might be completely different in the anaerobic conditions of the GIT. Earlier, we demonstrated various sensitivity of different strains of *E. coli* to oxygen that was connected with the activity of antioxidant enzymes (Semchyshyn et al., 2005b). Therefore, one may assume that the sensitivity of this bacterium to SFN could depend on the antioxidant potential and conditions of the experiment, particularly oxygen level. Interestingly, SFN effects on *E. coli* may be connected with blocking bacterial response to ROS at the level of master regulator OxyR possessing active thiol groups (Semchyshyn et al., 2005a) which potentially can be targeted by SFN.

Wang et al. (2023) found that SFN supplementation reversed the increases in the abundance of *Clostridium bolteae*, *Clostridium innocuum*, and *Clostridium symbiosum* in hyperuricemic rats. The latter bacterial species were positively associated with hyperuricemia. In addition, it significantly increased microbial diversity and altered their function, contributing to the treatment of hyperuricemic rats (Wang et al., 2023). Treatment with SFN for 12 weeks of autism spectrum disorders (ASD)-like rats and Chinese children also demonstrated positive therapeutic effects associated with the modulation of gut microbiota (Yang et al., 2023). In particular, network analysis identified 25 taxa associated with rat social behavior, eight of which were associated with SFN treatment of ASD-like rats. In addition, 35 changes in the abundance of gut microbiota that correlated with SFN treatment of ASD symptoms were found (Yang et al., 2023). In addition, sulforaphane reversed gut dysbiosis in mice with ulcerative colitis. After 7 days, intragastric administration of 20 mg/kg SFN did not significantly affect the composition of intestinal bacteria. However, after 14 days, SFN significantly increased the level of Bacteroidetes and decreased the level of Firmicutes changed by dextran sodium sulfate in mice with ulcerative colitis (Zhang et al., 2020). Thus, the consumption of both SFN and its biologically inert precursor GRP potentially can prevent dysbiosis contributing to the prevention/treatment of various physical and mental health disorders.

## 4 Molecular mechanism of action of dietary glucoraphanin/sulforaphane to maintain a healthy gut microbiota

**FIGURE 3:**
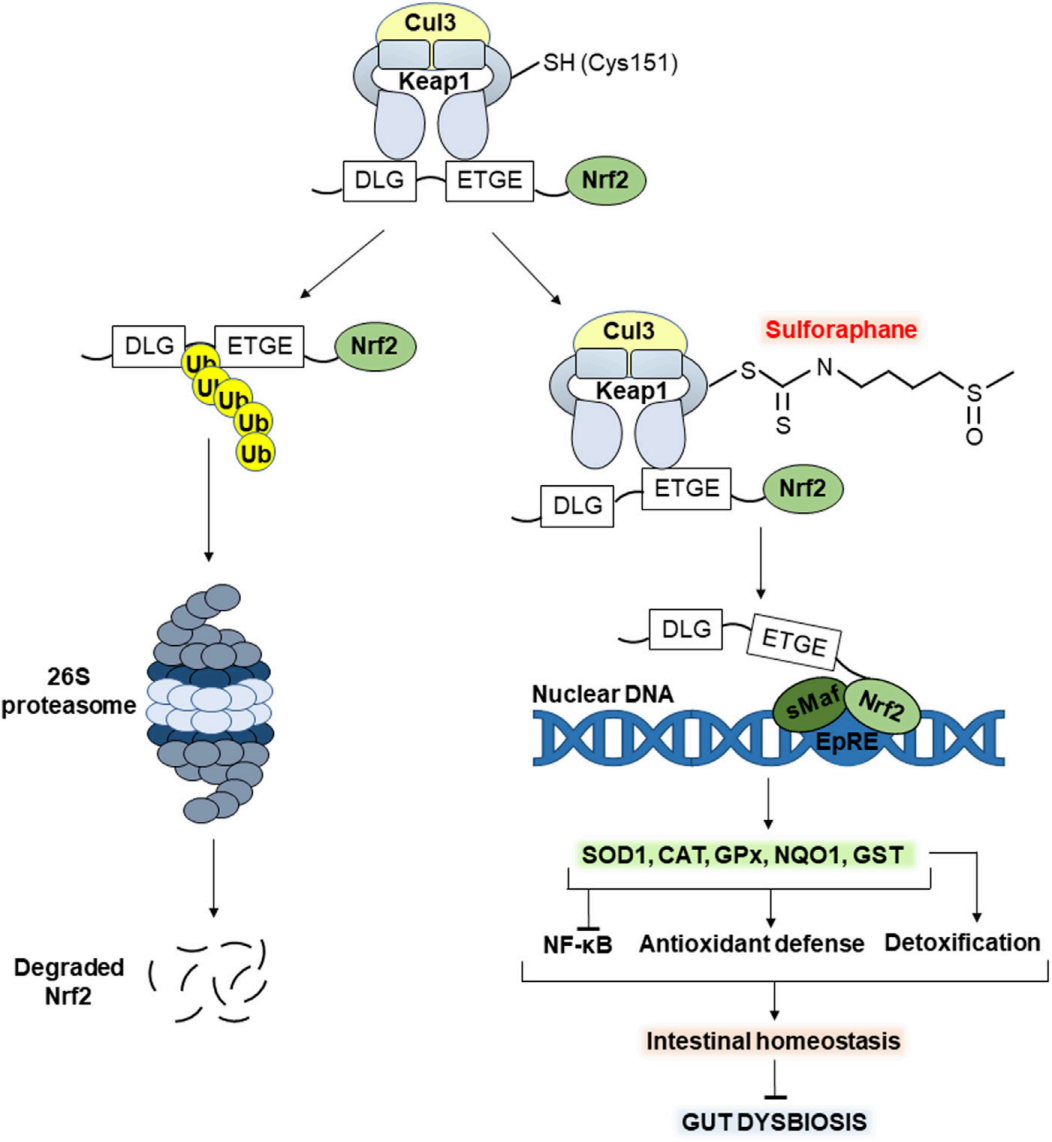
Molecular mechanism of action of sulforaphane (SFN). The electrophilic carbon of the isothiocyanate group of SFN interacts with the nucleophilic thiol group of Cys151 of the Keap1 protein, contributing to the activation of Nrf2 signaling. The latter increases the transcription of antioxidant enzymes such as SOD1, CAT, GST, GPx and NQO1. In turn, this enhances the potential of the antioxidant defense system, alleviating oxidative stress in the gut. In addition, increased Nrf2 signaling inhibits the transcription factor NF-κB associated with inflammation. Importantly, GST enzyme belongs also to phase II detoxification enzymes, contributing to the elimination of potentially toxic/toxic compounds for both the gut microbiota and the host in general. Together, all of the above maintain intestinal homeostasis preventing gut dysbiosis. See the text for details. Abbreviations: Cul3, cullin 3; Keap1, Kelch-like ECH-associated protein 1; Cys 151, SFN-sensitive cysteine residue; Nrf2, nuclear factor erythroid 2-related factor 2; DLG, ETGE, Nrf2 motifs for recognition/binding by Keap1; Ub, ubiquitin; sMaf, small musculoaponeurotic fibrosarcoma; EpRE, electrophile responsive element; NF-κB, nuclear factor κB; SOD1, superoxide dismutase 1; CAT, catalase; GPx, glutathione peroxidase x; NQO1, NAD(P)H quinone oxidoreductase 1; GST, glutathione-S-transferase.

The molecular mechanism of action of SFN in eukaryotes is well understood, but most intestinal microorganisms belong to prokaryotes. Therefore, here we focus on discussing the effects of SFN on their habitat, i.e., the gut, as a healthy gut microbiota is undoubtedly associated with gut health. Figure 3 schematically shows the molecular mechanism of action of dietary SFN, which prevents inflammation and oxidative stress effectively supporting intestinal homeostasis. Oxidative stress is a transient or long-term increase of the steady-state level of reactive oxygen species (ROS) leading to oxidative modifications of biomolecules and cell death via necrosis or apoptosis (Lushchak, 2014; Lushchak and Storey, 2021). The latter can potentially cause increased intestinal barrier permeability and associated gut dysbiosis. Whereas SFN is considered a potent activator of the Nrf2 (nuclear factor erythroid 2-related factor 2) signaling pathway controlling the cellular response to oxidative stress (Piotrowska et al., 2021; Dmytriv et al., 2024a).

Under homeostatic conditions, transcription factor Nrf2 is synthesized but constantly subjected to proteasomal degradation mediated by Keap1 (Kelch like ECH associated protein 1). In the cytoplasm, Keap1 homodimer forms ubiquitin E3 ligase complex with cullin 3 (Cul3), which polyubiquitinates Nrf2 protein leading to its continuous proteasomal degradation (Dmytriv et al., 2024a). Keap1 is the main negative regulator of Nrf2. It recognizes and binds the ETGE and DLG motifs in the structure of Nrf2 protein forming Keap1-Nrf2 complex. The first, ETGE, is high-affinity but exhibits slow rates of association/dissociation, whereas the second, DLG, binds faster, but with an approximately 100-fold lower binding affinity. In particular, the high-affinity ETGE is considered a hinge, anchoring Nrf2 to the Keap1, while DLG acts as a latch. Exposure to electrophiles such as SFN causes modification of several reactive cysteine residues of Keap1 with subsequent destabilization of the binding of Keap1-DLG (Shilovsky and Dibrova, 2023).

Takaya et al., (2012) found that point Cys151 mutation significantly reduced SFN-induced response to oxidative stress in mutant cells. In particular, the expression of some Nrf2 target genes was significantly abrogated. These results confirm that modification of Cys151 is key in the SFN-mediated activation of Nrf2 signaling (Takaya et al., 2012). Thus, SFN interacts with the thiol group of Cys151 leading to a partial loss of the Keap1-Nrf2 relationship (Figure 3, right). In turn, this prevents polyubiquitination and proteasomal degradation of Nrf2 protein stabilizing its levels (Yamamoto et al., 2018). Further Nrf2 translocates into the nucleus, heterodimerizes with one of the small musculo-aponeurotic fibrosarcoma (sMaf) proteins, binds to the antioxidant responsive element (ARE) or electrophile responsive element (EpRE) and promotes the transcription of genes encoding defense proteins/enzymes (Yamamoto et al., 2018). The latter include antioxidant enzymes such as superoxide dismutase 1 (SOD1), catalase (CAT), glutathione peroxidase (GP), GST, NAD(P)H quinone oxidoreductase 1 (NQO1), and others. Together, they neutralize ROS and their products of action, alleviating oxidative stress (Dmytriv et al., 2024b; Lushchak, 2011).

**FIGURE 4:**
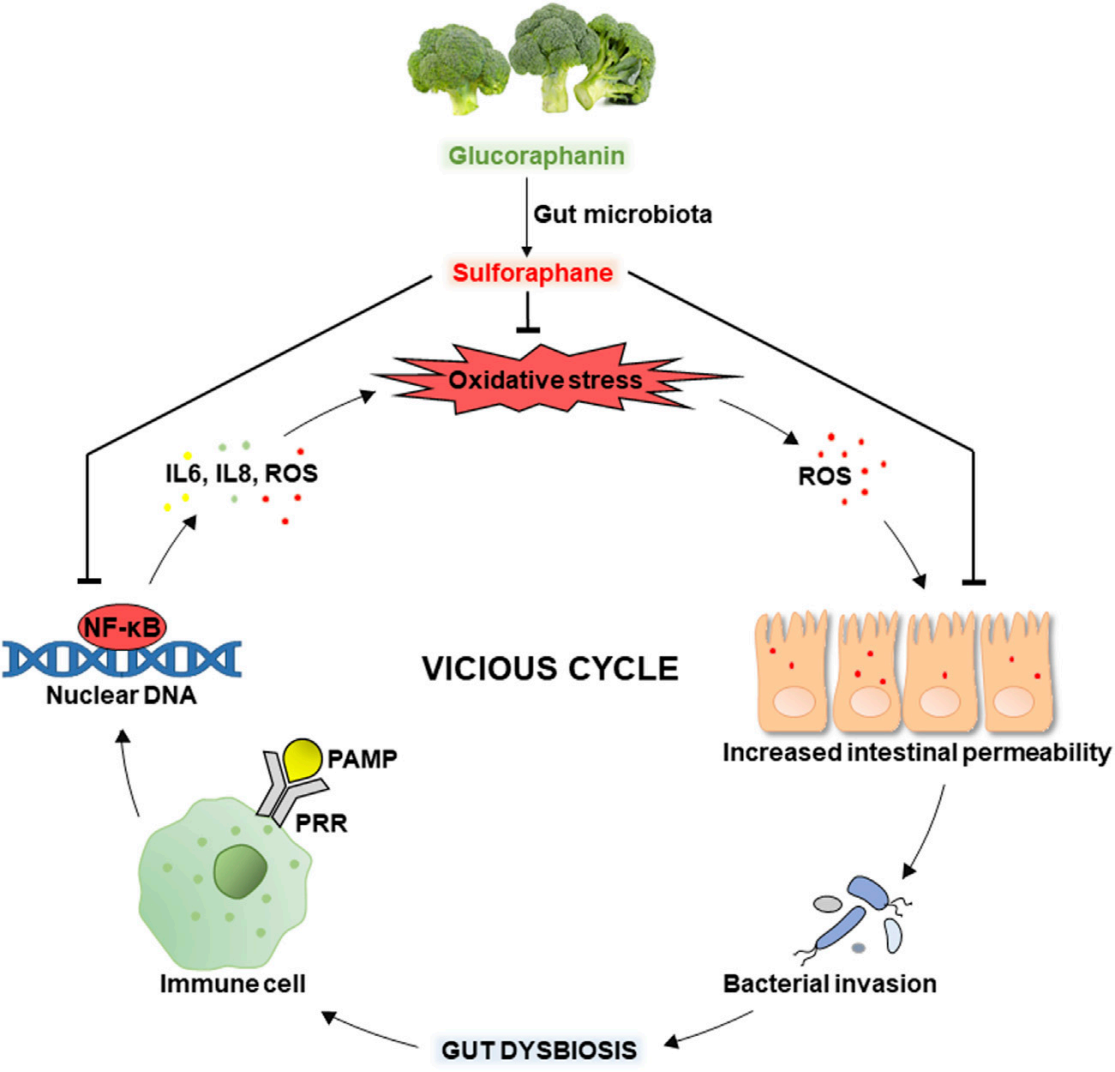
The vicious cycle of oxidative stress and gut dysbiosis: the role of sulforaphane.

Besides, SFN can potentially alleviate/break the vicious cycle of oxidative stress and gut dysbiosis shown in Figure 4. Intestinal oxidative stress associated with excessive ROS generation causes oxidative modifications to tight junction proteins leading to increased intestinal permeability, invasion of luminal bacteria, and gut dysbiosis. The latter can induce the inflammatory process related to recognizing bacterial pathogen-associated molecular patterns (PAMPs) by pattern recognition receptors (PRRs) of the host’s immune cells. This causes the activation of the molecular pathway NF-κB (nuclear factor κB), promoting the transcription of pro-inflammatory cytokines such as IL-6 and IL-8. In addition, activated immune cells significantly generate ROS, increasing oxidative stress (Dmytriv et al., 2024b) and closing the vicious cycle shown in the figure. While SFN inhibits oxidative stress, inflammation, and excessive intestinal permeability, alleviating the vicious cycle. Indeed, several studies show the effectiveness of SFN in improving antioxidant status and preventing oxidative stress in the gut mainly through activating Nrf2 signaling (Alattar et al., 2022; He et al., 2022; Ma et al., 2023).

Ma et al. (2023) established the protective role of SFN against oxidative stress induced by triphenyltin in *Cyprinus carpio haematopterus*. In particular, SFN improved antioxidant enzyme activities including SOD, CAT, and GPx, and also relieved the changes of inflammatory factors. In addition, SFN treatment caused a significant decrease in five inflammation-associated bacteria and normalized triphenyltin-induced changes in gut microbiota composition (Ma et al., 2023). In another study, SFN alleviated pro-inflammatory cytokine levels and increased tight junction protein expression in mice with dextran sulfate sodium (DSS)-induced ulcerative colitis. It also partially restored the disturbed gut microbiota composition caused by DSS administration, including changes in the relative abundance of Firmicutes, Bacteroidota, and Verrucomicrobiota (He et al., 2022). In addition, SFN treatment ameliorated colon and caecal mucosal epithelial damage in mice with N-butyl-N-(4-hydroxybutyl)-nitrosamine-induced bladder cancer through up-regulation of tight junction protein expression, downregulation of IL-6 release, and prevention of gut dysbiosis (He et al., 2018). Thus, the results described above may indicate that dietary SFN at least in part promotes a healthy gut microbiota by preventing the development of oxidative stress and inflammation in intestines. However, the question on selectivity of SFN effects on GIT microbiota is still open.

## 5 Conclusion and perspectives

Biologically inert GRP is a reserve form of SFN. The latter is chemically unstable due to the reactive carbon of the isothiocyanate group (N=C=S) that easily reacts with nucleophiles such as thiols. Thus, plants in a normal state practically do not contain SFN, but it is formed by hydrolysis of the GRP precursor by the enzyme myrosinase due to the destruction of plant tissues. In particular, chewing cruciferous vegetables rich in GRP leads to its release from vacuoles providing GRP availability for plant myrosinase leading to SFN formation.

Myrosinase is active at alkaline conditions in the oral cavity but inactivated in the stomach due to acidic conditions. In the intestine, GRP becomes available to the gut microbiota, which can metabolize it to SFN, SFN-nitrile, glucoerucin, erucin, or erucin-nitrile (Figures 1, 2). In particular, a positive association between members of the genus *Dorea*, *Bifidobacterium* and *R. torques* and SFN metabolite excretion was established. Genera such as *Lactococcus*, *Bifidobacterium*, *Lactobacillus*, *Bacteroides*, *Pseudomonas*, *Staphylococcus*, *Enterococcus*, and *Streptomyces* can potentially also exhibit myrosinase-like activity. Collectively, the composition, metabolic activity, and functionality of the gut microbiota significantly affect the metabolism of GLs. In particular, the individual microbial pattern can vary greatly between individuals, as can the level/composition of microbiota-produced GRP metabolites. However, as far as is known, consumption of dietary GRP enhances its microbial hydrolysis rates. Thus, regular consumption of cruciferous vegetables can potentially contribute to the formation of more significant concentrations of SFN. However, there is currently a significant gap in research on the microbiota-associated metabolism of GRP/SFN. For example, it is not clear whether intestinal microbiota can form SFN conjugates such as SFN-GSH or SFN-NAC. It is well known that adsorbed ITCs such as SFN and erucin enter the mercapturic acid pathway in the liver, forming N-acetylcysteine conjugates that are transported to the kidneys and excreted in the urine. Whereas the metabolism of SFN-nitrile and erucin-nitrile is poorly studied and needs more attention. In addition, some scientists overestimate the role of intestinal microbes in the metabolism of GLs and ITCs. However, some parts of GRP and SFN are not metabolized by gut microbiota and are excreted intact with urine or feces.

Both GRP and SFN may reciprocally modulate gut microbiota composition and functionality. In particular, effects such as increased gut microbiota richness and diversity and decreased Firmicutes/Bacteroidetes ratio have been reported, demonstrating the potential of ITCs for obesity prevention. In general, SFN exhibits both antimicrobial properties and can enhance the growth of some intestinal commensals or pathogens. It is worth noting that the gut microbiota lives in virtually anaerobic conditions of the GIT, so the results received under aerobic conditions (21% oxygen) may differ and even be opposite. This should be taken into account in future research. Another question is related to the selective effects of GRP and SFN in the gut microbiota to provide flowering of beneficial microorganisms. However, the molecular mechanisms are not sufficiently studied.

We hypothesize that the influence of SFN on the gut microbiota is at least partly related to the effects on their habitat, i.e., the gut such as (i) alleviation of oxidative stress and inflammation in the intestine, (ii) increase of the expression of tight junction proteins, preventing excessive intestinal permeability and invasion of luminal bacteria, and (iii) promotion of gut detoxification capability via increase of activity of phase II detoxification enzymes such as GSTs. In this way, SFN contributes to healthy gut microbiota, maintaining gut barrier integrity, intestinal redox homeostasis, and detoxification pathways. Overall, this demonstrates the clinical potential of SFN to prevent intestinal oxidative stress, inflammation, and gut dysbiosis, that can be used to develop new dietary approaches for gut health and overall wellbeing. At the same time, GRP/SFN are shown to be promising for the prevention of obesity and inflammatory bowel diseases. However, further studies are needed to elucidate the molecular mechanisms and the impact of individual microbial profiles on GRP/SFN metabolism. Additional attention should be paid to studying the metabolism of SFN-nitriles and other GRP derivatives, and their roles in the body.
